# Craniovertebral Junction Compression in Patients With Morquio Syndrome: Case Series and Literature Review

**DOI:** 10.7759/cureus.93079

**Published:** 2025-09-23

**Authors:** Purbaday Rakshit, Andi Sadayandi Ramesh, Rajasekar Gopikrishnan, Manoranjitha Kumari Mani, A Sathia Prabhu

**Affiliations:** 1 Department of Neurosurgery, Jawaharlal Institute of Postgraduate Medical Education and Research, Puducherry, IND

**Keywords:** cervical compression myelopathy, cervicomedullary junction compression, lysosomal storage disorder, morquio syndrome, mucopolysaccharidosis type iv

## Abstract

Mucopolysaccharidosis (MPS) is a group of lysosomal storage disorders characterized by defective degradation of glycosaminoglycans, resulting in progressive multisystem involvement. The central nervous system is most commonly affected, followed by the skeletal, cardiovascular, respiratory, and ophthalmological systems. MPS can cause craniovertebral junction (CVJ) stenosis due to the progressive accumulation of glycosaminoglycans (GAGs) in various connective tissues, including those of the spine. Cervical myelopathy is a serious and potentially reversible complication caused by odontoid hypoplasia, ligamentous thickening, and CVJ stenosis.

A retrospective case series of four paediatric patients, with a mean age of six years, who underwent neurosurgical intervention to relieve cervicomedullary compression (foramen magnum narrowing and C1 arch stenosis) at a single tertiary care center was conducted. Clinical presentation, relevant investigations, type of surgical management, and survival outcomes were reviewed through detailed analysis and follow-up data. Due to the limited sample size (N = 4), no formal statistical analysis was performed.

All patients showed preoperative clinical and radiological evidence of cervical cord compression. Postoperative outcomes were favorable in all four patients, with neurological improvement observed within six months of surgery. No major perioperative complications were reported. During follow-up (two to 68 months), three patients (75%) had no motor deficits, while one patient (25%) died following sudden respiratory distress at home five years post-surgery.

Cervical myelopathy in MPS patients requires early clinical suspicion and urgent imaging evaluation. Surgical decompression, either in the form of C1 posterior arch excision alone or combined with foramen magnum decompression, is effective in halting neurological progression and improving function. An individualized surgical plan based on preoperative imaging is therefore essential for optimal outcomes. Given the very small sample size, these findings should be considered exploratory and require confirmation in larger studies.

## Introduction

Morquio syndrome, or mucopolysaccharidosis type IV (MPS IV), is an autosomal recessive disease and a rare lysosomal storage disorder caused by a deficiency of enzymes involved in the degradation of glycosaminoglycans (GAGs), particularly keratan sulfate and chondroitin-6-sulfate [1-3]. There are two types of Morquio syndrome: type A, due to deficiency of N-acetyl-galactosamine-6-sulfatase (GALNS), and type B, due to deficiency of beta-galactosidase (GLB1). This enzymatic deficiency results in GAG accumulation within connective tissues, leading to a spectrum of skeletal abnormalities, most notably affecting the spine and craniovertebral junction (CVJ).

Hypoplasia of the odontoid process, periodontoid soft tissue deposition, and cervical stenosis contribute to myelopathy and quadriparesis in these patients. This progressive cervical myelopathy poses a significant threat to neurological function and overall quality of life. Despite its clinical importance, CVJ involvement in Morquio syndrome remains underrecognized.

There is limited consensus on the optimal timing and strategy for neurosurgical intervention in such cases. Early detection and timely management are critical to preventing irreversible neurological damage.

In this case series, we present our institutional experience with CVJ compression in patients with Morquio syndrome, detailing clinical presentation, radiological findings, surgical management, and outcomes. Existing literature shows that C1 cord compression in MPS IV patients under nine years of age is unusual and underreported. Hence, we aim to highlight the early onset of myelopathy, and our series contributes to the sparse literature on pediatric cervical myelopathy in MPS IV. We also provide a comprehensive review of the existing literature to emphasize the current understanding, challenges, and evolving treatment paradigms associated with this complex condition. Here, we present four cases of MPS type IV who underwent neurosurgical management and attempt to analyze their surgical outcomes.

## Case presentation

Data collection

**Figure 1:**
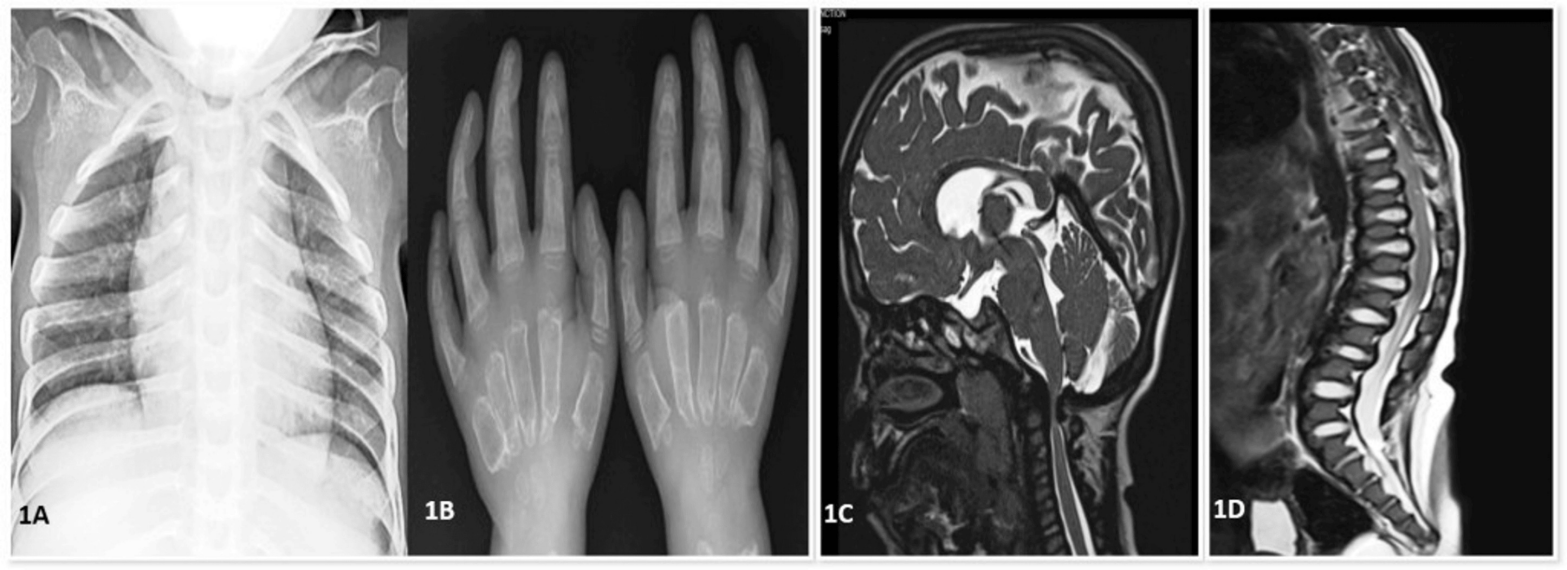
1A) Chest X-ray: Pectus excavatum deformity and short neck; 1B) Hand X-ray: diaphyseal thickening with short, wide metacarpals and dysplasia of the carpal bones, with increased scapholunar distance suggesting instability; 1C) MRI brain with cervical spine: craniosynostosis with scaphocephaly, bulging temporal squamae, small posterior fossa with the belly of the pons indented against the clivus, thickened retrodental ligaments, and reduced posterior atlantodental distance causing severe spinal canal narrowing with compressive myelopathy at C1 and the odontoid level; 1D) MRI thoracolumbar spine: vertebral bodies showing anterior beaking, prominent intervertebral disc spaces, and an increased angle between the sacrum and lumbar spine.

**Figure 2:**
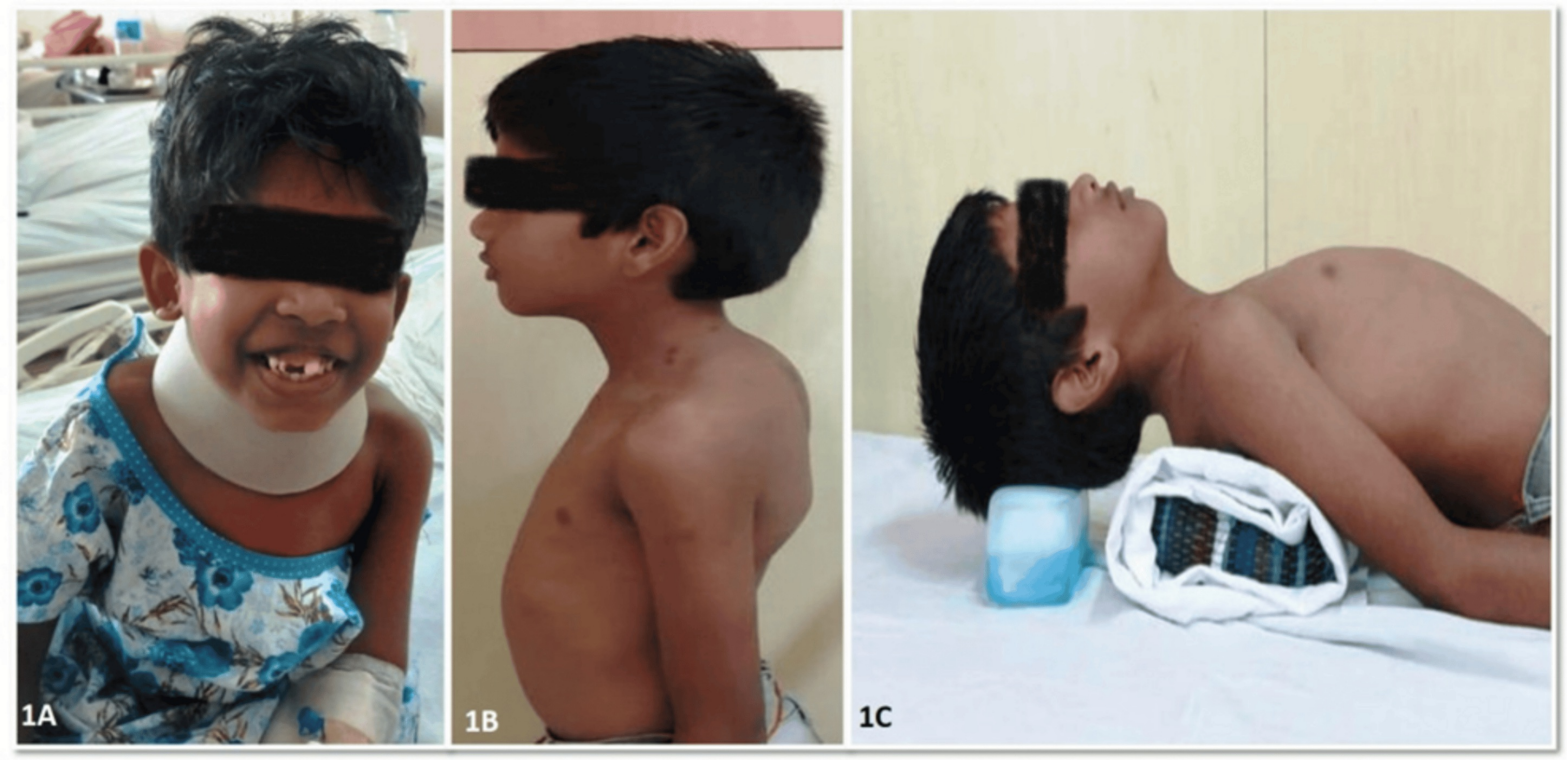
2A) A child with Morquio syndrome presenting with coarse facial features, including prominent upper incisors, widely spaced teeth, and a flattened nasal bridge. A cervical collar was required to stabilize the neck due to atlantoaxial instability caused by odontoid hypoplasia; 2B) A child with Morquio syndrome exhibiting a short trunk and neck, kyphotic deformity of the thoracic spine, pectus carinatum, and abnormal shoulder contour; 2C) Supine position demonstrating a fixed spinal deformity despite gravity, with pronounced thoracolumbar kyphosis, hyperextended neck, and short-trunk dwarfism.

This retrospective case series describes four cases of Morquio syndrome, in which the clinical presentation, radiological findings, surgical management, and outcomes were critically analysed. We included four pediatric patients (aged between two and nine years) diagnosed with MPS type IV who underwent neurosurgery at a tertiary care centre for cervical cord compression secondary to the disease. The surgeries were performed between 2018 and 2025, and follow-up data ranged from two to 68 months. The timing and type of surgery were decided after discussion with the pediatrics department, where the patients were initially evaluated. Detailed history taking, careful clinical examination, and relevant preoperative radiological findings (CT and MRI) demonstrating cord compression led to the surgical recommendation. Data collected included demographic information (age, sex, and relevant history), clinical findings, preoperative radiological findings, type of surgery, clinical outcomes, and survival status. Postoperative radiological findings were compared with preoperative findings and analysed. Due to the limited sample size (N = 4), no formal statistical analysis was performed. All patients exhibited characteristic features such as short stature, short neck, coarse facies, malar prominence, broad nasal bridge, and flat forehead. Craniosynostosis was present in one patient (Table 1 and Figures 1-2).

**Table 1:** List of mucopolysaccharidosis type IV cases. D: Dorsal spine; MPS: Mucopolysaccharidosis; CSF: Cerebrospinal Fluid; CTEV: Congenital Talipes Equinovarus; DTR: Deep Tendon Reflexes.

Cases	Age	Sex	Complaints	Clinical Findings	Pre-Operative Imaging (CT & MRI Spine Findings)	Post-Operative Imaging (MRI Findings)	Diagnosis	Type of Surgery	Outcome	Survival Status	Post-Surgery Follow-up (Months)
Case 1	5 y	F	Difficulty walking for 3 months, frequent falls while playing, weakness of both upper and lower limbs	Coarse facies, short stature, short stubby fingers, cloudy cornea, pectus excavatum, knock knees, brisk reflexes, low posterior hairline; child required assistance to walk, muscle strength equivalent to MRC grade 3	Foramen magnum stenosis with significant compression of the cervicomedullary junction, high T2 signal in the cord (myelomalacia), flattened vertebral bodies throughout the axial skeleton (platyspondyly), anterior central vertebral body beaking involving D12 vertebral body with focal kyphosis	Platyspondyly, cervicomedullary junction myelomalacia, no current compression of the spinal cord	MPS type IVA (Morquio) with high cervical myelopathy	Foramen magnum decompression with C1 posterior arch excision	Improvement in walking	Alive	41
Case 2	9 y	M	Failure to thrive, difficulty lifting hands above shoulders, flexion contractures of knees and elbows	Short stature, corneal clouding, pectus excavatum; motor strength grossly within normal limits	Anteriorly placed posterior arch of C1, cervical cord compression, odontoid peg hypoplasia, absent anterior and posterior arch in the midline of C1	Restoration of the thecal sac space at C1 with CSF flow, no cord compression, cord appears normal	MPS type IV (Morquio) with C1 arch stenosis	C1 arch excision	Motor improvement	Alive	68
Case 3	8 y	F	Upper limb weakness, inability to walk without support	Developmental delay, bilateral CTEV, coarse facies, short neck, short stature, paraparesis; with assistance, child was able to ambulate; muscle power MRC grade 3	J-shaped sella with mild shortening of clivus, reduced posterior atlantodental distance, severe spinal narrowing at C1 and odontoid level, platyspondyly, craniosynostosis, small posterior fossa with pons indented against clivus, non-ossification of anterior and posterior midline atlas arches, thickened retrodental ligaments	Postoperative changes with non-visualisation of posterior midline spinous process of C2, thickened retrodental ligaments, increased sagittal cord diameter	MPS type IVA (Morquio) with C1 myelopathy	C1 arch excision	Motor improvement postoperatively	Expired in 2023 (sudden respiratory distress at home)	-
Case 4	2 y	M	Gross motor delay, inability to walk without support	Child required assistance to walk; muscle strength equivalent to MRC grade 3; increased tone in all limbs, exaggerated DTRs, extensor plantar reflexes, short neck	Narrowing of the foramen magnum, cervical canal stenosis, J-shaped sella, flattened tuberculum sella	No compression of the cervical cord	MPS type IV (Morquio) with spinal canal stenosis	C1 arch excision	Motor improvement	Alive	2

Case 1

**Figure 3:**
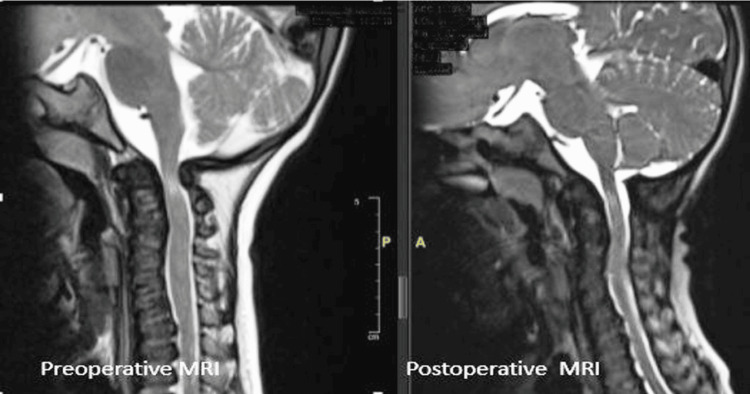
Case 1: Preoperative and postoperative MRI T2WI showing cervicomedullary compression with hypoplasia of the odontoid peg, and postoperative decompression of the spinal cord following surgery. T2WI: T2-Weighted Imaging.

A five-year-old female child diagnosed with MPS type IVA presented with complaints of difficulty walking and frequent falls over the preceding three months. On general examination, she was found to be severely underweight (10.6 kg, < -3Z) with stunted growth (84.8 cm, < -3Z). Physical examination revealed coarse facies, short stature, short stubby fingers, cloudy cornea, pectus excavatum, and knock knees. The child required assistance to walk, and muscle strength corresponded to MRC grade 3. Urinary GAG levels were elevated, and enzyme analysis confirmed galactose-6-sulfate sulfatase deficiency. X-ray revealed anterior beaking of vertebrae, bullet-shaped metacarpals, and oar-shaped lower ribs (Figure 1). MRI of the spine showed compression at the C1-C2 level; initially, the child did not demonstrate neurological deficits, but she later developed lower limb weakness with pyramidal signs and was admitted to Neurosurgery. MRI revealed foramen magnum stenosis with significant compression of the cervicomedullary junction, high T2 signal in the cord (myelomalacia), flattened vertebral bodies throughout the axial skeleton (platyspondyly) with posterior bowing of most vertebral bodies, associated indentation of the anterior thecal sac and spinal cord at multiple levels, and anterior central vertebral body beaking involving the D12 vertebral body with focal kyphosis (Figure 3). She underwent foramen magnum decompression with C1 posterior arch excision, which led to resolution of symptoms. Postoperative MRI showed adequate spinal cord decompression. At her current three-year follow-up, she has shown significant neurological improvement.

Case 2

**Figure 4:**
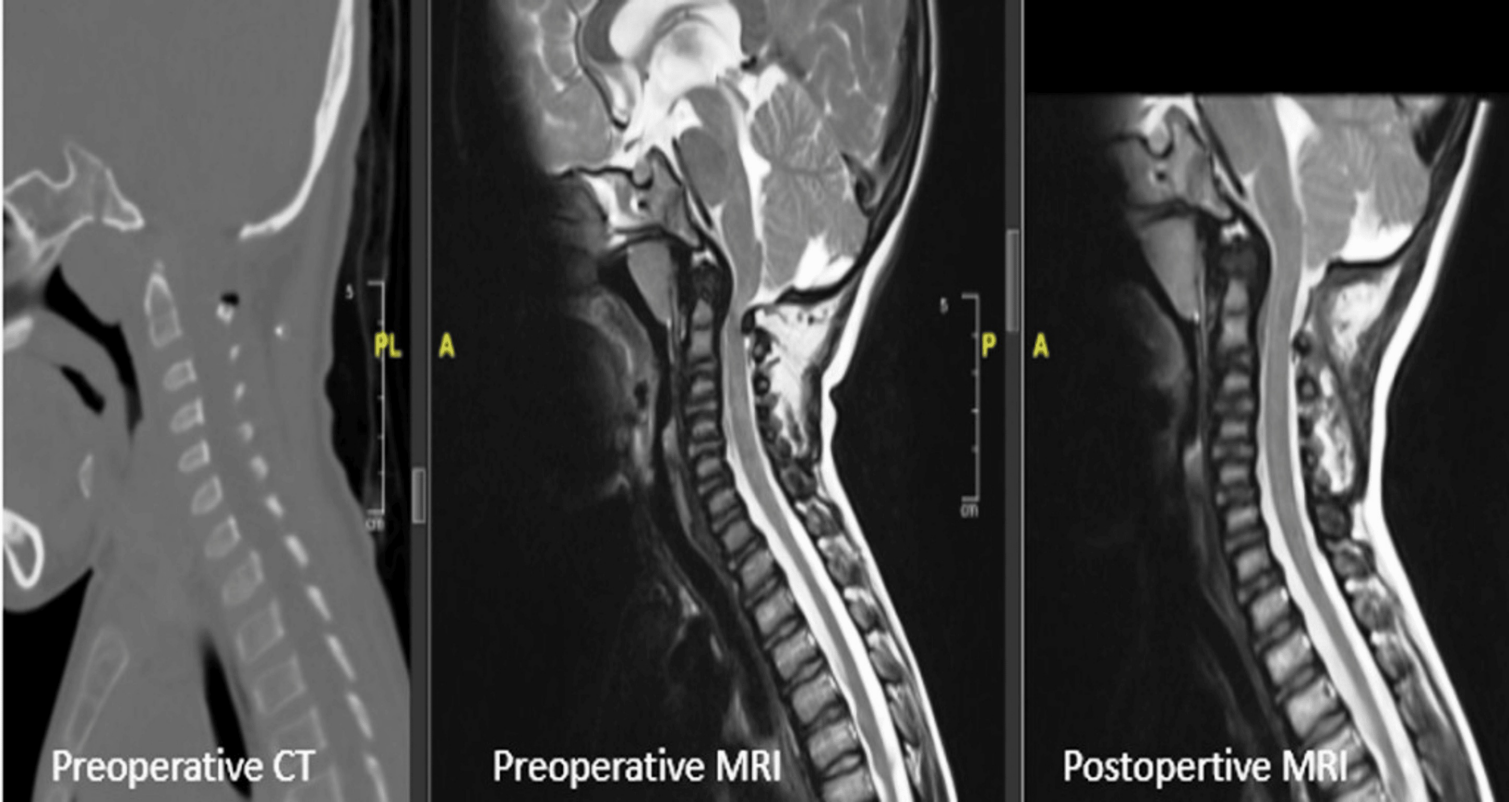
Case 2: Preoperative and postoperative CT and MRI images of the cervical spine showing adequate cervicomedullary decompression.

A nine-year-old male child, diagnosed with MPS type IV, presented with complaints of short height and failure to thrive. On physical examination, he had increased tone in all four limbs, though motor strength was grossly within normal limits. Muscle wasting and deformities were noted in the bilateral wrists and knees. MRI of the spine revealed bony defects in the midline of the C1 vertebra anteriorly and posteriorly, with the anteriorly placed posterior arch of C1 causing narrowing of the posterior thecal sac. The posterior elements of the C1 vertebra were compressing the cervical cord, and hypoplasia of the odontoid peg was also noted (Figure 4). He underwent excision of the posterior arch of C1 as well as release of the constricting band below the arch. Postoperative MRI showed restoration of the thecal sac space at the level of C1 with evidence of CSF flow. His neurological symptoms progressively improved, and at the current six-year follow-up, he has no neurological deficits.

Case 3

**Figure 5:**
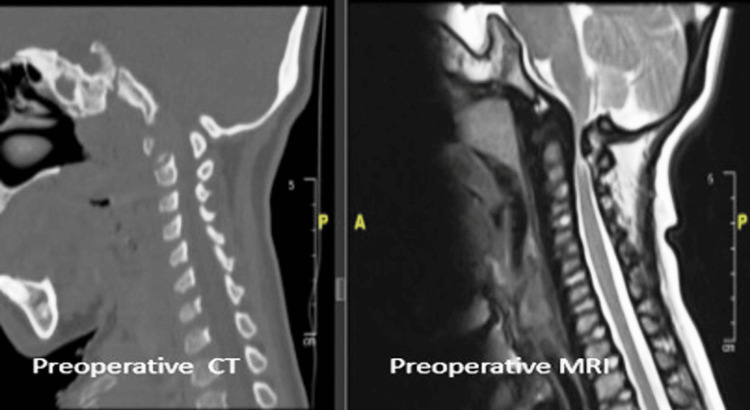
Case 3: Preoperative CT and MRI images of the cervical spine showing cervicomedullary compression and platyspondyly (decreased vertebral body height with increased anteroposterior diameter). AP: Anteroposterior.

An eight-year-old female child, diagnosed with MPS type IV, presented with bilateral upper limb weakness and inability to walk without support for the past two months. On physical examination, she was found to have developmental delay, CTEV of bilateral feet, coarse facial features, short neck, and short stature. With assistance, she was able to ambulate, and muscle power was graded as MRC grade 3. Spiral CT of the spine showed a J-shaped sella with mild shortening of the clivus and reduced posterior atlantodental distance, causing severe spinal narrowing and platyspondyly. MRI of the spine revealed craniosynostosis with scaphocephaly and bulging temporal squamae due to fused sagittal and coronal sutures, congenital variation with non-ossification of anterior and posterior midline atlas arches, thickened retrodental ligaments, and reduced posterior atlantodental distance causing severe spinal canal narrowing at C1 and the odontoid level. Platyspondyly with anteroinferior beaking of vertebral bodies was also noted (Figure 5). She underwent C1 posterior arch excision and showed improvement in symptoms. According to her parents, she had an episode of sudden respiratory distress at home five years post-surgery, following which she could not be revived.

Case 4

**Figure 6:**
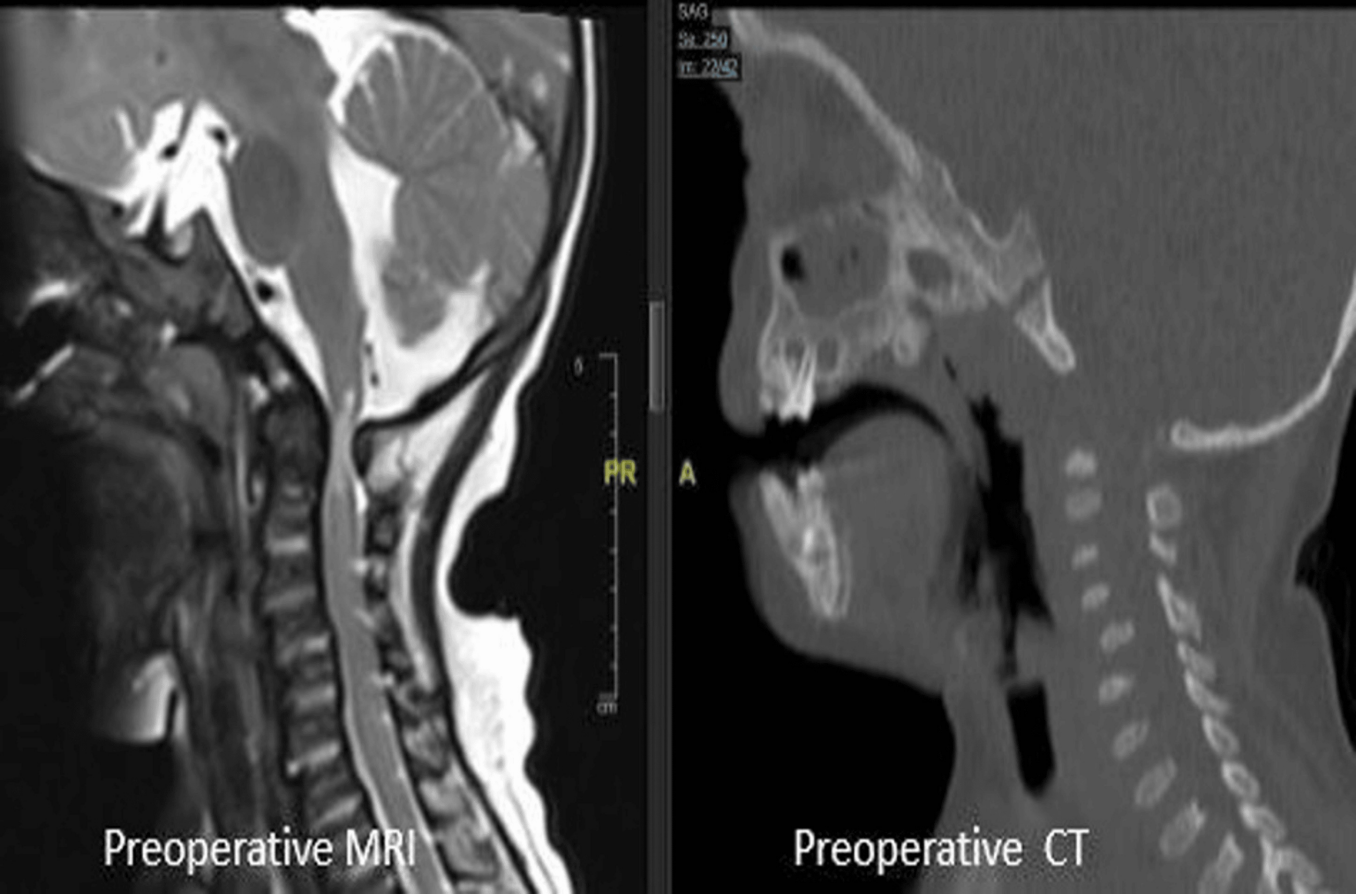
Case 4: Preoperative CT and MRI images of the cervical spine showing severe cervical stenosis.

A two-year-old male child, diagnosed with MPS type IV, presented with inability to walk without support and gross motor developmental delay for the past six months. His mother also reported a rapid increase in head circumference. On physical examination, the child required assistance to walk, and muscle strength corresponded to MRC grade 3. Deep tendon reflexes were exaggerated, lower limbs had hypotonia, and plantar reflexes were extensor. Bowing of the lower limbs was also noted (Figure 6). MRI of the brain showed narrowing of the foramen magnum with cervical canal stenosis, cervicomedullary compression, a J-shaped sella with flattened tuberculum sella, and a prominent suprasellar cistern. He underwent excision of the C1 arch and fibrous end below the arch. At the current two-month follow-up, he is ambulant without support and has no neurological deficits.

Surgical management

Preoperative planning included detailed imaging review with spiral CT and MRI of the cervical spine, along with screening of the whole spine. Decompression was performed under general anaesthesia in the prone position for all cases. Patients were either positioned on a horseshoe support or fixed in a head frame.

Excision of the posterior arch of the C1 vertebra was performed in all four patients, while one patient additionally required foramen magnum decompression along with C1 arch excision due to severe narrowing of the foramen magnum. Thick fibrous bands were identified below the posterior arch of C1, causing spinal cord constriction in all four patients; these bands were excised. The surgical goal was to achieve adequate decompression of the spinal cord at the cervical level. Fusion was not attempted in any of these patients.

Postoperatively, patients were monitored in the neurosurgical intensive care unit and placed in a hard cervical collar for 48 hours for immobilisation. The postoperative period was uneventful in all cases, and outcomes were favourable, with gradual improvement in preoperative weakness of the upper or lower limbs. Postoperative imaging (MRI cervical spine) confirmed adequate decompression of the cord.

Results

**Figure 7:**
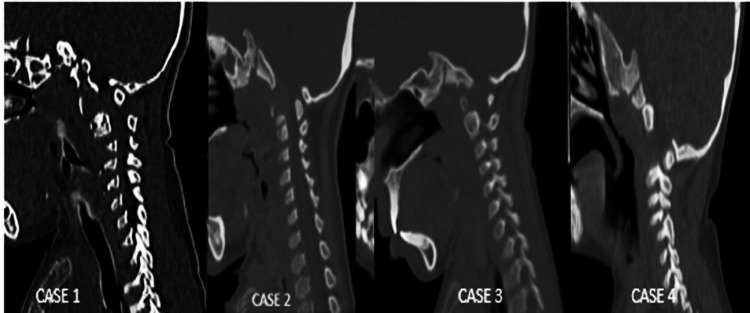
Preoperative CT of the cervical spine showing a thickened posterior arch of C1 in all patients.

Four patients, with an average age of six years (range: two to nine years) and a sex ratio of 1:1 (two males and two females), underwent cervical decompression for compressive myelopathy, as summarised in Table 1. Preoperative clinical signs of myelopathy were documented for all patients. There were no intraoperative or postoperative complications in any of them. At discharge following surgery, all patients showed postoperative improvement in walking and muscle strength and were discharged home. At the most recent follow-up (ranging from two to sixty-eight months), three patients were doing well, while one patient died five years post-surgery following sudden respiratory distress at home. Despite resuscitation efforts, she could not be revived, though there were no preceding complaints. We observed thickened posterior arches of C1 in the preoperative CT spine images of all patients, which appeared to be significant (Figure 7). The arches were not only thick but also flat in contour, with a central cartilaginous midline portion. We also noted that the C1 posterior arch lay in very close proximity to the foramen magnum. This anatomical relationship made surgical excision of the posterior arch considerably more difficult and underscores the need for utmost care to avoid injury to the marginal sinus (sinus marginalis) and the dura mater at the foramen magnum.

## Discussion

Mucopolysaccharidosis (MPS) constitutes a group of inherited lysosomal storage disorders caused by deficiencies in enzymes responsible for the degradation of glycosaminoglycans (GAGs). This enzymatic deficiency results in the accumulation of partially degraded GAGs within lysosomes, leading to multisystem involvement, including skeletal abnormalities, organomegaly, visual and auditory impairments, and, in some subtypes, neurological deterioration. Among the various subtypes, MPS type IV (Morquio syndrome) is particularly characterized by severe skeletal dysplasia without significant cognitive impairment, and often presents with cervical spine instability or stenosis, which can lead to potentially life-threatening spinal cord compression. The incidence is similar in males and females, with estimates ranging from 1 in 75,000 to 1 in 200,000 live births. MPS type IV is further classified into subtypes based on the specific enzymatic deficiency: MPS type IVa, IVb, and IVc. Clinically, MPS types IVa and IVb are similar, though both display wide heterogeneity.

Despite the clinical variability, the syndrome is typically associated with coarse facial features and multiple skeletal dysplasias, such as pectus carinatum, knock knees, keel thorax, short stature, genu valgus, flat foot, coxa valga, gait disorders, and spinal abnormalities including kyphosis, platyspondyly, scoliosis, wedge or ovoid vertebrae, and cervical spine anomalies. Importantly, while intellect is preserved, neurological symptoms, such as weakness, motor delays, gait abnormalities, and, in severe cases, quadriparesis, may develop due to cervical spinal cord compression [4-8]. In the current series, all four patients demonstrated neurological manifestations ranging from gross motor delay and frequent falls to upper limb weakness and paraparesis. These clinical features were consistent with cervical myelopathy confirmed on imaging. MPS type IV can be differentiated from other types of MPS by the absence of intellectual disability. Biochemical studies and genetic evaluation are essential for diagnostic confirmation in these patients.

Diagnosis is confirmed by direct enzyme assay on fibroblasts or leukocytes obtained from fibroblast cultures or heparinized blood. Mutations in the GALNS or GLB1 genes must be identified to differentiate between type A and type B Morquio syndrome. Radiological evaluation plays a pivotal role in the diagnosis and surgical planning of MPS-associated spinal pathology. Classical imaging features include narrowing of the foramen magnum, hypoplasia of the odontoid process, anterior and posterior arch anomalies of the atlas (C1), and severe spinal canal narrowing at the cervicomedullary junction. MRI remains the modality of choice for assessing cord compression and myelomalacia, whereas CT scans provide superior detail of the bony anatomy. In our cohort, all patients demonstrated C1-level stenosis, with additional findings such as thickened retrodental ligaments, platyspondyly, J-shaped sella, and congenital atlas arch anomalies, reflecting the diverse skeletal manifestations of Morquio syndrome [9-12]. Airway assessment using imaging is also essential before intubation, as these patients frequently present with obstructive symptoms. Three-dimensional tomographic reconstructions of the spine and trachea are particularly useful in evaluating respiratory symptoms and planning intubation in these patients.

Symptomatic treatment depends on the age group at which the diagnosis is established. Complications of thoracic and spinal deformities are more evident up to six years of age. The constitution of a multidisciplinary medical team involving an otolaryngologist, orthopedist, and pulmonologist is beneficial for such patients. Cardiological evaluation is essential, as cardiovascular manifestations can appear beyond one year of age. Skeletal abnormalities are most evident in patients older than six years, for whom decompression of the cervical spine is usually recommended to prevent and treat myelopathy. Early surgical intervention is often indicated in symptomatic patients or those with significant imaging abnormalities. Indications for surgery include progressive neurological deficit, atlantoaxial instability, and spinal cord compression. The mainstay of treatment includes posterior decompression via C1 arch excision, often combined with foramen magnum decompression where necessary, with or without fusion [13]. In this series, all four patients underwent C1 arch excision, with one also undergoing extended foramen magnum decompression due to significant cervicomedullary compression. Pediatric spine surgery, however, carries significant challenges owing to small anatomy, bony fragility, instrumentation difficulties, and anaesthetic risks related to airway management due to short necks and soft tissue infiltration in MPS.

Surgical outcomes were generally favorable, with three patients demonstrating clear postoperative improvement in motor function or ambulation. Radiologically, decompression was confirmed by restoration of CSF flow and alleviation of cord compression. However, postoperative cervical spine MRI could not be performed in two patients due to the difficulty of undergoing MRI under general anaesthesia in unstable conditions. One patient, unfortunately, succumbed to sudden respiratory distress, underscoring the fragility of this patient population and the importance of vigilant postoperative monitoring and follow-up.

In 2014, the FDA approved elosulfase alfa for the treatment of MPS type IVA. This is a recombinant formulation of the lysosomal enzyme N-acetylgalactosamine-6-sulfatase [14]. Some improvement has been reported in patients treated with this therapy. However, no enzyme replacement therapy is currently available for MPS type IVB. Reports indicate that patients with mild phenotypes may survive into the seventh decade of life, often maintaining normal height and continued growth into adolescence. In contrast, survival in severe forms usually does not exceed 25 years, with death most commonly resulting from cardiac or respiratory complications.

## Conclusions

Morquio syndrome presents with a distinct constellation of skeletal and neurological manifestations, with cervical spine involvement being a major contributor to morbidity. Early recognition through clinical evaluation and imaging, followed by timely surgical decompression, can significantly improve outcomes in affected individuals. Postoperative outcomes in patients with MPS-associated cervical myelopathy largely depend on preoperative neurological status, timing of intervention, and the extent of spinal compression. In this small series, the majority of patients had favorable outcomes, with improvement in motor function and stabilization of neurological symptoms. However, delayed diagnosis or surgical intervention, along with airway and respiratory complications, continues to contribute to morbidity and mortality.

The case of the deceased patient highlights the importance of multidisciplinary perioperative care, including respiratory support and early recognition of complications. Genetic counseling and early diagnosis in resource-limited settings are also crucial. Given the complex systemic involvement in MPS, long-term follow-up and comprehensive multidisciplinary management are essential to optimize quality of life and survival in these patients.
